# Rosetta in CAPRI rounds 13–19

**DOI:** 10.1002/prot.22784

**Published:** 2010-06-01

**Authors:** Sarel J Fleishman, Jacob E Corn, Eva M Strauch, Tim A Whitehead, Ingemar Andre, James Thompson, James J Havranek, Rhiju Das, Philip Bradley, David Baker

**Affiliations:** 1 Department of Biochemistry, University of WashingtonSeattle, Washington 98195; 2 Department of Genome Sciences, University of WashingtonSeattle, Washington 98195; 3 Department of Genetics, Washington University School of MedicineSt. Louis, Missouri 63110; 4 Department of Biochemistry, Stanford UniversityStanford, California 94305; 5 Computational Biology Program, Fred Hutchinson Cancer Research CenterSeattle, Washington 98109; 6 Howard Hughes Medical Institute (HHMI)Seattle, Washington 98195

**Keywords:** CAPRI, structure prediction, protein-protein interactions, RNA-protein interactions, Rosetta, flexible-backbone modeling, conformational changes, docking, backrub, fragment insertion

## Abstract

Modeling the conformational changes that occur on binding of macromolecules is an unsolved challenge. In previous rounds of the Critical Assessment of PRediction of Interactions (CAPRI), it was demonstrated that the Rosetta approach to macromolecular modeling could capture side chain conformational changes on binding with high accuracy. In rounds 13–19 we tested the ability of various backbone remodeling strategies to capture the main-chain conformational changes observed during binding events. These approaches span a wide range of backbone motions, from limited refinement of loops to relieve clashes in homologous docking, through extensive remodeling of loop segments, to large-scale remodeling of RNA. Although the results are encouraging, major improvements in sampling and energy evaluation are clearly required for consistent high accuracy modeling. Analysis of our failures in the CAPRI challenges suggest that conformational sampling at the termini of exposed beta strands is a particularly pressing area for improvement. Proteins 2010. © Wiley-Liss, Inc.

## INTRODUCTION

The conformations of biological macromolecules often change on complex formation with binding partners. These changes are in many cases key to understanding specificity and energetics of binding events. Correct modeling of structural adaptation on binding is a prerequisite to atomic-accuracy modeling of molecular interactions. However, the energetic differences associated with changes in backbone configurations can be subtle, and the space of even local conformational perturbations when combined with alternative docking arrangements is vast, making the accurate modeling of conformational changes on binding extremely challenging.

The Rosetta approach to macromolecular modeling (reviewed in Ref.^1^) consists of two stages: (1) fast low-resolution structural diversification followed by (2) all-atom refinement of candidate models using an energy function that is dominated by van der Waals, hydrogen bonding, and solvation terms. The native state of the system is expected to be a low-energy conformation within a landscape of higher energy non-native states. Accordingly, in several of the computational protocols described below, we used rounds of Monte Carlo-based backbone sampling starting from the unbound components to generate alternatives that were subsequently docked using RosettaDock.^2^ We then used further rounds of backbone remodeling in the bound state to relax the system. Models were ranked according to total system energy and by computed binding energy.^3^

We found that when backbone changes are modest and the rough configuration of the native complex is known, this approach results in low-energy docked configurations that are in close agreement with the crystal structure. More extensive backbone remodeling, such as by fragment insertion,^1^ produced low-resolution improvements, even for an RNA case. Nevertheless, in cases where there is significant backbone movement we have not achieved the atomic-accuracy configurations needed for robust docking, underscoring the importance of continued development of methods for the estimation of the energetics of binding and of conformational sampling at the interface.

## METHODS AND RESULTS

### T30: Rnd1–GTP binding to plexin B1

**Figure 1:**
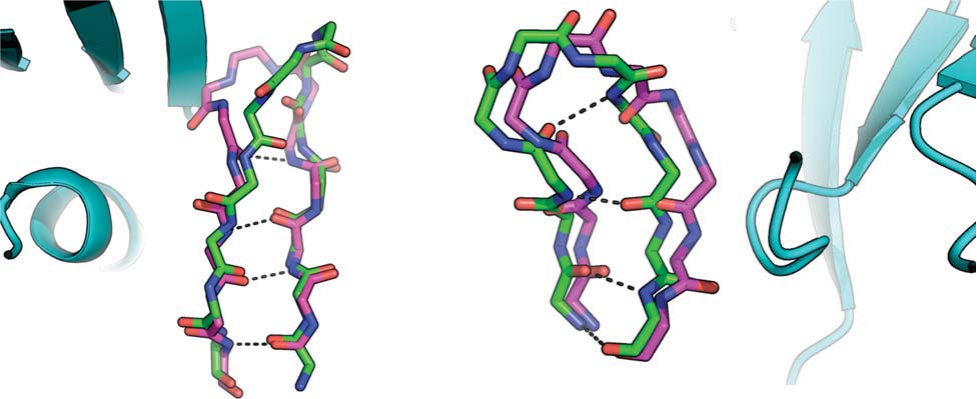
Overlay of unbound (magenta) on bound (green) structures showing backbone deformations in interfacial β-strands. Backbone–backbone hydrogen bonds are marked as dashed lines. Bound partners are in cyan. Left: plexin from T30. Right: centaurin-alpha from T39. All figures were generated using PyMol.^5^

The contributors provided a structure of plexin in its unbound conformation. Starting from this structure, we tried several modeling strategies involving different extents of remodeling of the plexin loops. The most conservative strategy involved eliminating an extended loop that plexin uses for homodimerization [encompassing residues 1822–1834 according to the numbering in Protein Data Bank (PDB) entry 2REX^4^] followed by fixed-backbone docking to identify low-energy conformations. Another strategy eliminated the loop encompassing residues 1790–1800 on plexin. We also tried docking the two partners after rebuilding loops on plexin using fragment insertion. Each strategy was attempted using a plexin monomer or dimer separately. Unfortunately, none of the submitted models were of acceptable quality. Comparing the crystal structure of the bound complex with the starting unbound monomers, there are significant changes in the backbone conformation of one of the β-strands that is involved in forming the binding pocket on plexin (Fig 1, left). We did not take into account remodeling of the secondary-structural elements, and redocking any of our models on the bound pose results in severe van der Waals clashes. This modeling attempt and another below points to the pliability of edge β-strands in transitioning between bound and unbound states and the importance of accounting for such deformations in docking simulations.

### T33 and T34

Targets 33 and 34 offered extensive tests of many aspects of the Rosetta macromolecule modeling framework beyond macromolecule docking, from protein homology modeling to small-molecule docking to RNA *de novo* modeling. We describe each of these aspects in turn.

#### Protein and cofactor homology modeling

As suggested in the T33 target description, the RNA methyltransferase was modeled by homology to the supplied crystallographic structure of a related methyltransferase. We made use of the Rosetta “loop-relax” protocol, previously tested in CASP6^6^ and CASP7,^7^ as implemented on the Rosetta@Home distributed computing network.

Because of uncertainties in the position of the *S*-adenosyl methionine (SAM) cofactor, this small molecule was not included during the refinement. Instead, the SAM moiety was transformed to adopt a binding mode similar to that in the ErmC′ structure (PDB entry: 1QAO^8^); full-atom refinement was performed under the Rosetta energy function augmented with restraints that preserved hydrogen bonding and packing interactions, analogous to those observed for ErmC′. The current unavailability of the crystallographic structure for T33 prevents us from assessing the accuracy of this small-molecule remodeling.

#### RNA modeling

**Figure 2:**
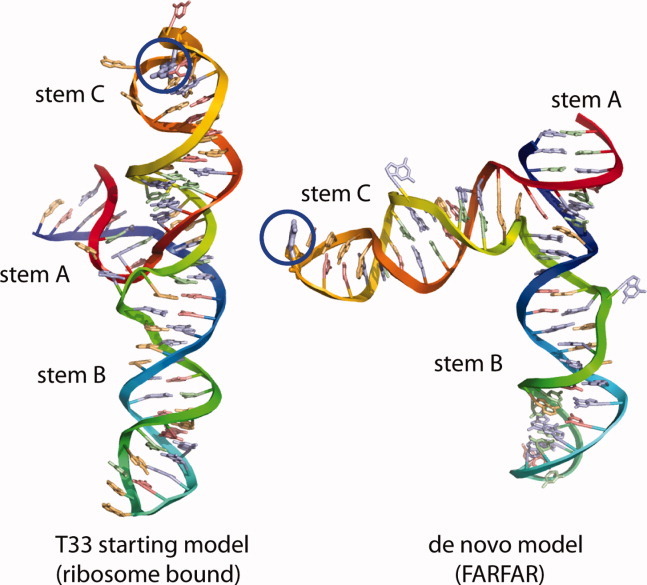
Models of the 74-nucleotide RNA transcript containing three stems A, B, and C from the RNA provided in CAPRI target T33; the unbound conformation as provided by CAPRI organizers (left) has a different helical arrangement from *de novo* models generated by FARFAR (right). The interaction partner for this target was a methyltransferase protein that modifies the RNA residue circled in blue.

For target T33, modeling the RNA component of the bound complex provided a novel challenge in CAPRI. Published biochemical data suggested that the RNA’s conformation differs in solution compared with the supplied ribosome-bound structure or the desired methyltransferase-bound structure. We, therefore, applied a preliminary version of fragment assembly of RNA with full-atom refinement (FARFAR^9^) to model and then assemble the noncanonical subpieces of the RNA. One of these pieces, a junction between three helices (marked as A, B, and C in Fig 2), is the central determinant of the global fold of the RNA. In the supplied ribosome-bound structure, helices B and C form a colinear arrangement [Fig 2(A)]. Unfortunately, FARFAR modeling of this junction did not converge at atomic resolution. Nevertheless, at lower resolution, the final low-energy configurations shared an overall global fold that was distinct from the ribosome-bound fold. Helices A and B were predicted to be colinear, and helix C (containing the methylation site) formed an ∼60° angle with helix B [Fig 2(B)].

Tests docking these models to our protein homology models with experimental restraints (see below) further supported the *de novo* RNA fold compared with the ribosome-bound RNA fold. Release of the protein-bound RNA crystallographic model revealed that the predicted conformational rearrangement of the three helices occurs. Our model attained nucleotide resolution accuracy over the junction: 5.4 Å over C4′ atoms, compared with 12.4 Å in the previously available ribosome-bound conformation. Nevertheless, uncertainties in fine base-pairing details, amplified by the lever-arms of the helical extensions, led to an accuracy over the entire RNA (10.6 Å root mean square deviation (RMSD)) that was too low to enable even “acceptable” quality protein–RNA docking.

During modeling, it was also clear that fine features of the hairpin C containing the methylation site were likely incorrect. For example, our de novo models were in excellent agreement with the NMR solution structure of this piece, but neither set of models could explain published chemical accessibility measurements of the protein/RNA complex,^10^ presumably reflecting a conformational rearrangement stabilized by protein contacts. Furthermore, our preliminary docked models suggested that the methylation site-containing residue should have been rotated by 180° to make contact with the modeled SAM ligand; this rotation appears to be enabled by the loop C conformational change. More powerful RNA loop-modeling algorithms developed since CAPRI round 14 appear to more readily sample the structure, properly displaying the methylation site. We look forward to further CAPRI and CASP RNA challenges to test these RNA-modeling methods.

#### Additional restraints

In addition to the SAM/RNA methylation site contact, we inferred looser restraints based on published biochemical experiments, falling into four classes. Set 1 included atoms for which experimental evidence suggested contacts with protein groups (or RNA conformational rearrangements on binding). These restraints came from dimethyl sulfate (DMS) protection and phosphorothioate-interference studies. Set 2 included atoms, which were accessible to DMS methylation in the bound protein/RNA complex. Sets 3 and 4 were assumed to be either within ∼4 Å or greater than 4 Å from a protein atom, based on published NMR chemical shift analysis. These experimental data were converted to soft distance restraints for use during the low-resolution docking simulations. In addition, a weak bonus was given for protein–RNA contacts involving conserved residues in the methyltransferase.

#### Protein–RNA docking

To generate candidate docked conformations for the protein–RNA complex, we used a directed sampling approach that reflected the strong orientational restraints between the SAM molecule and the RNA substrate. Monte Carlo perturbations and rigid-body minimization were performed in the six internal coordinates linking the RNA with the SAM molecule (these degrees of freedom comprised one bond length, two bond angles, and three bond torsions). This was done within Rosetta by defining a pseudobond connection between the SAM and the RNA, linking the Cε atom of the SAM and the N1 atom of the modified base on the RNA. At the start of each docking simulation, these six internal coordinate degrees of freedom were randomized within relatively generous ranges that reflected the uncertainty in the positioning of the SAM and for target 33, the uncertainty in the RNA internal conformation.

Low-resolution docking simulations were then performed by making small random perturbations to these six degrees of freedom, in the context of Rosetta’s standard low-resolution potential augmented by two generic protein–RNA interaction terms, a soft clash term, and bonuses for favorable residue interactions with the RNA backbone. In addition, we applied specific interaction restraints derived from the literature, described above. The resulting low-resolution models were clustered to identify common modes of interaction, and a subset of the clusters were refined in a more realistic all-atom potential. To allow for flexibility in the conformation of loops that were built during the homology modeling, a subset of the docking simulations were performed with trimmed protein structures from which poorly conserved loops had been deleted; these loop regions were then rebuilt in the context of the bound RNA after low-resolution docking.

The final submitted models combined examples of both approaches: the top-ranked model (in order of submission and as judged by similarity to the target structure) was generated using this trim-dock rebuild strategy, whereas model 2 (which was almost as accurate) was built by full-structure docking. In the end, model ranking and selection were primarily guided by consensus, after the low-resolution modeling simulations. Given the uncertainties in the precise structure of the variable regions of the methyltransferase and in the SAM orientation, we were hesitant to use the all-atom energy after refinement as a quality metric. Instead, the all-atom refinement helped to generate physically realistic final structures for submission.

### T37: Arf6 binding to the LZ2 domain of JIP4

The structure of the second leucine-zipper domain of JIP4 was modeled using a combination of *de novo* and rigid-body docking methods. We previously developed fold-and-dock^11^ to predict the structure of symmetrical homo-oligomers directly from protein sequence. Fold-and-dock combines the *de novo* folding and symmetrical assembly^12^ methods in Rosetta and has been shown to successfully predict the structures of many small homomeric assemblies, including coiled-coils.^11^ In T37 fold-and-dock primarily generated antiparallel coiled-coils, whereas the biological data suggested a parallel orientation. We, therefore, forced the parallel orientation by placing single helices from the lowest energy fold-and-dock models in a parallel orientation based on the rigid-body relationship between chains in the GCN4 leucine zipper,^13^ which was suggested as a template by the organizers. The orientation of the two helices was then optimized using the symmetrical-assembly method to remove unfavorable interactions. The lowest energy model was subjected to an all-atom refinement where rigid-body, backbone, and side chain degrees of freedom were simultaneously optimized followed by another round of refinement using the symmetrical-assembly method. Comparison of the submitted model and the JIP4 domain in the crystal structure (PDB entry 2W83^14^) verified the success of *de novo* modeling with a Cα RMSD of 1.8 Å between model and crystal structure over the 120 residues present in both structures, and an all-atom RMSD of 2.3 Å. The ends of the coiled-coil are modeled less precisely, largely due to a divergence from two-fold symmetry in these regions of the crystal structure. This result confirms, together with previous results,^11^ that *de novo* prediction of the structure of homodimeric coiled-coils can be routinely achieved at high accuracy.

For the macromolecular docking of JIP4 with Arf6, we employed global and local docking based on biochemical data on the binding mode. Each scheme involved a sequence of RosettaDock,^2^ backrub,^15^ and minimization over rigid-body, side chain, and backbone degrees of freedom. The best-energy model from global docking produced a configuration that involved mainly the switch I region of Arf6 and overlapped with the guanosine triphosphate (GTP)-binding site. Comparing with the crystal structure, this model captures 90% of the interface residues on JIP4 according to the organizers (it is shifted in register by one turn compared with the native state). Additionally, we used local docking to focus on the epitope around the hydrophobic triad (residues F47, W62, and Y77), which is exposed in GTP-bound Arf6^16^ and is a common effector-binding site for the Arf protein family.^17^, ^18^ Our best scoring models using this approach involved the amino-terminal region of JIP4 in binding. Most interface residues of Arf6 overlap with our prediction. However, in our model, Arf6 is flipped by 180° relative to the crystal structure, which is unsurprising considering the inherent symmetry of a coiled coil. This error underscores the importance of intensive sampling of degrees of freedom that are compatible with the internal symmetry axes of the constituent molecules.

### T38 and T39: Centaurin-alpha 1 and the FHA domain of KIF13B

For target 38, we were given the unbound domain of the centaurin-alpha 1 protein and the sequence of the FHA domain. We built models of the FHA domain by generating alignments to template structures using HHSearch^19^; 10,000 models were then built using the Rosetta rebuild and refine protocol.^20^ We then selected the best 1000 models by Rosetta energy. The low-energy models were clustered and the largest two clusters contained >70% of the models. The cluster centers were then used as inputs for docking against the centaurin-alpha 1 domain in PDB entry 3FEH.

Comparing these models of centaurin-alpha 1 homology modeling with the subsequently released crystal structure (PDB entry: 3FM8) shows that these models reasonably preserved many features of the templates; however, there were some inaccuracies in the models. In particular, we copied the loop spanning residues 453–464 (numbering according to PDB entry 3FEH) from the templates due to the high-confidence alignment between this segment and the templates. In retrospect, the differences in the bound structure of this loop were significant, pointing out that it should have been built *de novo*. The best-ranked entry in our submission was model 10 with ligand RMSD of 4.6 Å (Table I). Comparing the model with the subsequently released crystal structure shows that while the model clearly captures the correct surface on KIF13B, the atomic details of the interaction are completely wrong.

**Table I:** Summary of Performance in CAPRI Rounds 13–19

Target	Receptor–ligand	Type^a^	I_RMSD (Å)^b^	L_RMSD (Å)^b^	F_nat^b^	#h/m/a/i^c^
T29	Trm8/Trm8 tRNA guanine-N(7) methyltransferase	B–U	9.2 (8)	26.5 (4)	0.04 (8)	0/0/0/10
T30	Rnd1/Rho GTPase binding domain (RBD)	U–U	45.7 (3)	16.7 (3)	0.00	0/0/0/10
T33	RNA/protein	H–H	21.0 (1)	14.2 (1)	0.09 (8)	0/0/0/10
T34	RNA/protein	B–H	1.5 (1)	1.7 (1)	0.49 (3)	0/4/2/4
T37	Arf6/LZ2	U–H	9.8 (1)	26.8 (4)	0.00	0/0/0/10
T38	Centaurin/FHA	U–H	4.6 (1)	18.7 (10)	0.20 (10)	0/0/0/10
T39	Centaurin/FHA	U–B	10.2 (3)	18.4 (3)	0.08 (5)	0/0/0/10
T40	Trypsin/inhibitor	U–B	0.5 (1)	1.4 (1)	0.81 (1)	3/0/0/0

The rank of the model in our submission is shown in parentheses.

a Starting structures: B, bound; U, unbound; H, homology unbound.

b Measures according to CAPRI assessors (http://capri.ebi.ac.uk). I_rmsd, interface backbone rmsd; L_rmsd, ligand backbone rmsd; F_nat, fraction of native contacts.

c Counts of models according to accuracy: h, high; m, medium; a, acceptable; i, incorrect.

In T39, we started from the bound structure of the forkhead-associated domain (FHA) domain provided by the contributors and an unbound structure of centaurin (PDB entry 3FEH). Using these structures, we conducted docking simulations^2^ followed by backrub backbone motions^15^ and minimization of backbone, side chain, and rigid-body degrees of freedom. Unfortunately, none of the submitted models was deemed of acceptable quality. In retrospect, a centaurin interfacial beta sheet undergoes significant backbone remodeling to form the binding site for the FHA domain in the unbound to bound transition (Fig 1 right). None of our models showed remodeling of this kind, and so this binding site remained cryptic in our simulations. We note that most of our submitted models exhibited larger buried surface areas than the crystal structure (averaging 1740 Å^2^, compared with 1300 Å^2^ in the native interaction). A bias toward larger buried surface areas might be an artifact of allowing backbone motions after docking.

### Target 40: API-A and Trypsin

The bound structure of the double-headed arrowhead protease inhibitor-A (API-A) was provided by the contributors, together with an unbound structure of bovine trypsin (PDB entry 1BTY^21^). Though API-A is known to simultaneously bind two trypsin molecules (information provided by organizers), the challenge was to predict only one of the binding modes. As API-A was provided in the bound state, the initial orientation of API-A relative to trypsin was determined by comparison with the known structures of other trypsin-inhibitor complexes.^22^ Each positively charged residue of API-A was superimposed on Arg515 of the bovine pancreatic trypsin inhibitor (BPTI) complex (PDB entry 1EJM^22^). Only one residue, API-A Lys145, was capable of making a homologous interaction without causing extensive clashes. Additionally, Lys145 is hosted on an API-A surface loop that is highly similar to the BPTI loop. The API-A orientation obtained through superimposition was therefore used for an extensive local search.

We noted that this placement of API-A resulted in several clashes with the unbound trypsin model. Because these clashes were spatially minor, though energetically severe, we opted to forego extensive backbone remodeling, and instead focus on subtly optimizing the interface of the complex. Trypsin interface positions within 8 Å of API-A were subjected to backrub^15^ backbone perturbations, followed by local Monte Carlo-based docking with rounds of side chain packing and minimization.^2^ Promising trajectories from this combined backrub/docking protocol were minimized over rigid body, side chain, and backbone degrees of freedom. Final models were selected based on total system energy, calculated binding energy,^3^ and recapitulation of active rotamers within the trypsin catalytic triad.

**Figure 3:**
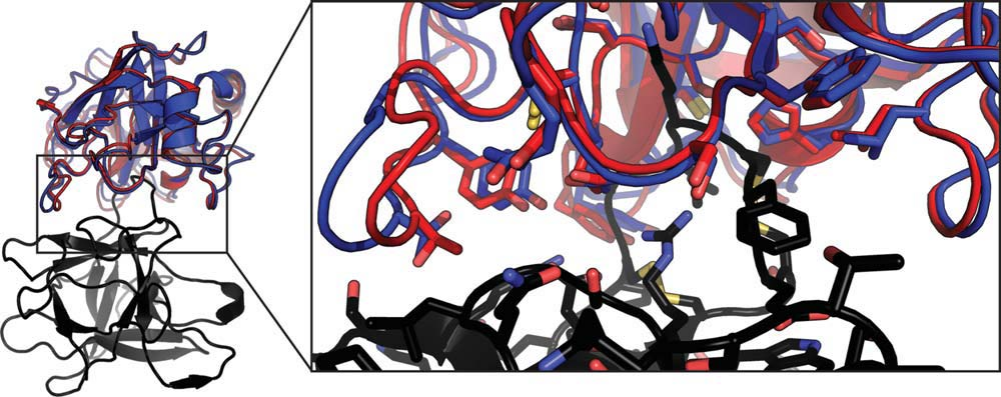
Comparison of the crystal structure of API-A (black) bound with trypsin (red) with the nearest to native submitted model (blue). Residues within 4 Å of the interface are shown in sticks.

The best model submitted for T40 has an interface RMSD of 0.5 Å and total ligand RMSD of 1.4 Å to the solved crystal structure of API-A bound to trypsin (PDB entry: 3E8L;^23^ Fig 3). Although this is clearly a correct solution, the small differences between the submitted model and the crystal structure highlight challenges for docking with backbone flexibility. The unbound structure of trypsin is closer to the bound structure (ligand Cα RMSD 0.2 Å) than any model submitted by any group, including ours, indicating that accurately modeling backbone flexibility remains a challenging problem, even when backbone changes are minor. However, as noted above, docking the unbound structure itself introduces many clashes, most of which were relieved by using the backrub protocol. Inspection of unsubmitted docking trajectories, which display total ligand RMSD’s of as little as 0.5 Å but relatively high system energies, indicates that the backrub protocol may have deviated from the crystal structure to relieve interfacial strain within the bound API-A model. Despite these minor shortcomings, T40 represents a successful application of flexible backbone docking, in that major interfacial clashes introduced by the unbound model were relieved via backbone moves to yield favorably scoring, near-native solutions.

### T41 and T42

We did not participate in this round because our group collaborated with the contributing teams.

## CONCLUSIONS

In these rounds of CAPRI, a main focus for our group was modeling backbone conformational changes induced by binding. Some of the results, particularly for protein/RNA modeling (see below), are very encouraging. For protein–protein docking, our results show important areas for future improvement, in particular, the aforementioned deformation of exposed β-strands on binding, which we have not taken into account in these rounds.

We are grateful for opportunities to blindly test a number of new approaches from the Rosetta community. For example, the RNA/protein targets T33 and T34 tested our ability to simultaneously sample small molecule, protein, and nucleic-acid components, a problem we could only recently start to tackle, because of our transition to an object-oriented framework. It was gratifying to have achieved a low-resolution prediction of the RNA fold (in T33), and the leading, medium-resolution predictions for the entire complex (in T34). However, because the full crystallographic model of this target has not been released, it is difficult for us to precisely delineate what problems will need the most development to enable future improvements. There are likely many areas to fine-tune, as these problems were remarkably complex, including homology modeling of both macromolecule partners and docking of three components (protein, RNA, and cofactor). We look forward to more multicomponent prediction problems in future rounds of CAPRI.
